# Magnesium Oxide Use and Clinical Outcomes in CKD Patients: Evidence from a Nationwide Population-Based Cohort Study in Taiwan

**DOI:** 10.7150/ijms.125059

**Published:** 2026-03-17

**Authors:** Po-Jen Hsiao, Liam Li-An Tsou, Chung-Chi Yang, Li-Yen Huang, Ruei-Lin Wang, Jenq-Shyong Chan, Kun-Lin Wu, Yung-Hsi Kao, Chu-Lin Chou

**Affiliations:** 1 Division of Nephrology, Department of Internal Medicine, Tri-Service General Hospital, National Defense Medical University, Taipei, Taiwan.; 2 Division of Nephrology, Department of Internal Medicine, Taoyuan Armed Forces General Hospital, Taoyuan, Taiwan.; 3 Department of Life Sciences, National Central University, Taoyuan, Taiwan.; 4 Division of Biochemistry, Department of Chemistry, Hofstra University, Hempstead, New York, USA.; 5 Division of Cardiovascular Medicine, Taoyuan Armed Forces General Hospital, Taoyuan, Taiwan.; 6 Cardiovascular Division, Tri-service General Hospital, National Defense Medical University, Taipei, Taiwan.; 7 School of Medicine, National Tsing Hua University, Hsinchu, Taiwan.; 8 Institute of Bioinformatics and Structural Biology, National Tsing Hua University, Hsinchu, Taiwan.; 9 Division of Nephrology, Department of Internal Medicine, School of Medicine, College of Medicine, Taipei Medical University, Taipei, Taiwan.; 10 Division of Nephrology, Department of Internal Medicine, Landseed International Hospital, Taoyuan City, Taiwan.

**Keywords:** acute kidney injury, acute kidney disease, chronic kidney disease, end-stage renal disease, cardiac arrhythmia, myocardial infarction, magnesium oxide

## Abstract

**Background:**

Magnesium homeostasis in chronic kidney disease (CKD) is complex, and serum magnesium concentrations reflect only approximately 1% of total body magnesium. Both magnesium deficiency (hypomagnesemia) and excess (hypermagnesemia) have been linked to adverse cardiovascular outcomes, a concern that is particularly relevant in patients with CKD. Magnesium oxide (MgO) is frequently prescribed for dyspepsia and constipation in clinical practice; however, its clinical impact in CKD patients remains uncertain and warrants further investigation.

**Materials and methods:**

We included non-dialysis CKD patients identified from the Taipei Medical University Clinical Research Database (TMUCRD) between 1998 and 2021. Adherence to MgO was assessed using the medication possession ratio (MPR). The primary outcomes were acute kidney injury (AKI), acute kidney disease (AKD), hospitalization for AKI, end-stage renal disease (ESRD) requiring dialysis, congestive heart failure with pulmonary edema, cardiac arrhythmia, and acute myocardial infarction. Baseline comorbidities assessed prior to the index date included hypertension, diabetes, hyperlipidemia, ischemic heart disease (IHD), ischemic stroke, congestive heart failure, atrial fibrillation (AF), peripheral arterial disease (PAD), chronic obstructive pulmonary disease (COPD), chronic liver disease (CLD), and dementia. These variables, along with relevant medications, were included as covariates in multivariable models to adjust for potential confounders.

**Results:**

Before matching, 6,105 MgO users and 10,143 non-users were identified; approximately 73% of MgO users had MPR <40%. In the ACE inhibitor (ACEI)/angiotensin receptor blocker (ARB) and pre-end-stage renal disease (pre-ESRD) program cohort, 207 MgO users and 1,401 non-users were included; after matching, 151 MgO users and 302 non-users remained. Dementia was more prevalent among MgO users, whereas diabetes was more common in non-users. MgO use was associated with higher risks of AKI, AKD, ESRD requiring dialysis, cardiac arrhythmia, and myocardial infarction in both unmatched and matched cohorts. In matched CKD patients, adjusted hazard ratios (aHRs) were 37.0 for AKI, 6.26 for AKD, 3.13 for ESRD, 2.06 for cardiac arrhythmia, and 1.86 for acute myocardial infarction. In the matched ACEI/ARB and pre-ESRD cohort, MgO users also demonstrated higher risks of AKI (aHR = 16.1) and AKD (aHR = 2.79). Cumulative incidence analyses consistently showed worse outcomes among MgO users. Among MgO users, advancing CKD stage was associated with progressively higher risks of adverse outcomes, particularly in stages 4-5. Both unmatched and matched analyses demonstrated a dose-response pattern, with the highest hazards observed for dialysis progression and cardiac arrhythmia.

**Conclusion:**

In this large cohort study, MgO use in CKD patients was associated with increased risks of AKI, AKD, ESRD, arrhythmia, and myocardial infarction. The magnitude of risk appeared greater in advanced CKD stages. These findings highlight the importance of careful risk-benefit assessment and close clinical monitoring when prescribing MgO in this high-risk population.

## Introduction

The kidneys play a central role in regulating magnesium balance by adjusting urinary excretion according to systemic magnesium status, ranging from 0.5% to 70% of filtered magnesium. Approximately 80-90% of magnesium reabsorption occurs through passive, paracellular pathways in the proximal tubule and thick ascending limb of Henle's loop, whereas only 5-10% is actively reabsorbed in the distal convoluted tubule. Magnesium homeostasis in chronic kidney disease (CKD) is complex. With progressive loss of renal function, as seen in advanced CKD and dialysis-dependent patients, there is an increased risk of magnesium accumulation and toxicity 1-3. Conversely, conditions such as malnutrition, concurrent diuretic or proton pump inhibitor use, and dialysis with low-magnesium dialysate may predispose patients to hypomagnesemia 4-8. Magnesium deficiency in CKD has been associated with higher parathyroid hormone levels, elevated systolic blood pressure, and increased cardiovascular risk. Kanbay M, et al. reported that CKD patients with serum magnesium levels below 2.05 mg/dL had significantly higher cardiovascular mortality 8. Importantly, the clinical scenarios of hypomagnesemia may contribute to endothelial dysfunction, vascular stiffness, arteriosclerosis, and adverse cardiovascular events, which are the leading causes of mortality in the CKD population 9-14.

There is currently no definitive method for assessing magnesium status. In clinical practice, a combination of dietary history, serum magnesium, and urinary magnesium excretion is often used, although each approach has important limitations 15. Given its diverse physiological functions, magnesium is essential in the prevention and management of numerous diseases. Hypomagnesemia has been linked to several chronic conditions, including Alzheimer's disease, insulin resistance and type 2 diabetes mellitus, hypertension, cardiovascular disease (such as stroke), migraine, and attention deficit hyperactivity disorder (ADHD) 16. Dietary assessment, whether through food diaries or recall of magnesium-rich food intake, is inherently inconsistent due to its retrospective design, regional variability in food processing, and temporal changes in nutrient composition. Regular updates to food composition databases are therefore essential for accurate estimation of dietary magnesium intake 16.

Magnesium oxide (MgO) is widely prescribed in clinical practice for the management of constipation, dyspepsia, and occasionally hypophosphatemia due to its laxative and antacid properties. The use of MgO as a laxative is particularly prevalent in Taiwan and Japan, whereas in many Western and other international settings, osmotic agents such as polyethylene glycol or lactulose are more commonly employed as first-line therapies for constipation 17. In patients with CKD, magnesium metabolism is often dysregulated, and both deficiency and excess can have important clinical implications. While MgO is commonly used in clinical practice, particularly for gastrointestinal indications and hypomagnesemia, its effects on magnesium balance and the broader impact on renal and cardiovascular outcomes in patients with advanced stages of CKD remain uncertain. Serum magnesium levels are influenced by multiple factors, including renal clearance, dietary intake, concomitant medications, and comorbidities, making interpretation in CKD particularly complex 13-16. Although dysmagnesemia has been linked to increased risks of vascular calcification, cardiovascular events, and mortality, direct evidence regarding the impact of MgO use on clinical outcomes in CKD patients remains limited. Clarifying this relationship is crucial for optimizing treatment strategies and reducing potential complications in this high-risk population.

## Materials and methods

### Data source

We used the Taipei Medical University Clinical Research Database (TMUCRD) for this study. The TMUCRD contains electronic medical records of 4.1 million patients from three affiliated hospitals, which are Taipei Medical University Hospital, Wanfang Hospital, and Shuang Ho Hospital from 1998 to 2021. All basic information, cause of death, medical information, cancer registry, diagnosis, treatment procedure and laboratory test result of the participants were available for analysis.

### Study design

In this study, the exposure group were those using magnesium oxide (MgO) and the control group were those never used MgO. The index date was defined as the index date of magnesium oxide use. We included the CKD patients not receiving dialysis in this study. A random date after the diagnosis of CKD was assigned to the control group as the index date. MgO users and non-users were matched according to sex, age, comorbidities, and medications by propensity score matching in 1:1 ratio. Patients who were younger than 30 years, and whom without a minimum of 2 years of data available following were excluded. We further investigated the cohort of CKD patients with receiving ACE inhibitors (ACEIs)/angiotensin receptor blockers (ARBs) and pre-end-stage renal disease (pre-ESRD) program. 1:2 propensity score matching was applied to obtain the matched cohort.

Patients were eligible for the program if they met the following criteria: the primary outcomes were AKI, AKD, ESRD with dialysis, cardiac arrhythmias, congestive heart failure with acute pulmonary edema, and acute myocardial infarction. Furthermore, in this study, the primary endpoints, which represented AKI-AKD-ESRD in the progression of dialysis, were those defined in the ADQI 18 and KDIGO 19 workshops. AKI was defined as an abrupt decrease in kidney function that occurred within 7 days or less after the index date and was divided into stages 0, 1, 2, and 3 multiplied by the serum creatinine level. AKD was described as acute or subacute damage and loss of kidney function for a duration of 7-90 days after exposure to an AKI episode and was divided into stages 0, 1, 2, and 3 multiplied by serum creatinine levels. All the serum creatinine levels included in the analysis were chosen on the basis of the respective highest values obtained within 0-7 and 7-90 days for AKI and AKD, respectively. ESRD with dialysis was defined as an order code by the National Health Insurance. In addition, multidisciplinary medical teams, including nephrologists, health education nurses, and nutritionists, provided comprehensive medical assessments, laboratory examinations, and patient education every three months. Those enrolled in the program were also cared for according to applicable clinical guidelines at different stages of CKD 20. The date of initial enrolment in the program was treated as the index date.

### Main outcome and covariates

The primary outcomes were acute kidney injury (AKI), acute kidney disease (AKD), hospitalization for AKI, ESRD requiring dialysis, congestive heart failure with pulmonary edema, cardiac arrhythmia, and acute myocardial infarction. Serum variables including creatinine, estimated glomerular filtration rate (eGFR), hemoglobin, and albumin were included as adjustment covariates. Baseline comorbidities assessed prior to the index date included hypertension, diabetes, hyperlipidemia, ischemic heart disease (IHD), ischemic stroke, congestive heart failure, atrial fibrillation (AF), peripheral arterial disease (PAD), chronic obstructive pulmonary disease (COPD), chronic lung disease and dementia. Concomitant medications considered were antiplatelets, warfarin, rivaroxaban, angiotensin-converting enzyme inhibitors/angiotensin receptor blockers (ACEI/ARB), β-blockers, calcium channel blockers (CCBs), statins, and antidiabetic agents, including metformin, thiazolidinediones, sulfonylureas, alpha-glucosidase inhibitors (AGIs), dipeptidyl peptidase-4 inhibitors (DPP4), and insulin. Patients were classified as MgO users if they had a pharmacy claim for MgO in combination with ACEIs/ARBs within three months of study initiation. Medication adherence was assessed using the medication possession ratio (MPR), calculated as the proportion of days covered by dispensed prescriptions during a specified interval. Participants who discontinued therapy for over 90 days during follow-up were censored and excluded from further analysis. MPR was categorized as <40% (poor adherence) and ≥80% (high adherence). An 80% threshold is commonly considered clinically meaningful, reflecting consistent medication availability with minimal treatment gaps 21, 22.

### Statistical analysis

We summarized the number and percentage for categorical variables, and the mean and standard deviation for continuous variables. The chi-square test and Student's T-test were applied to assess the difference between two groups. The univariable and multivariable Cox proportional hazard model was used to obtain the risk of outcome. The cumulative incidence curves were plotted by the Kaplan-Meier method and tested by the log-rank test. All analyses were conducted using SAS software, version 9.4 (SAS Institute Inc., Cary, NC). A significant level was p-value less than 0.05. The incidence per 100 person-years of outcomes was calculated via Kaplan‒Meier estimation. A Cox proportional hazards regression model was used to evaluate the hazard ratio (HR) for risk outcomes associated with MgO, after controlling for demographic and clinical factors 23. Patients who died, were lost to follow-up, or were discharged before the event of interest occurred during the follow-up period were censored.

## Results

### Baseline characteristics of CKD patients

**Figure 1:**
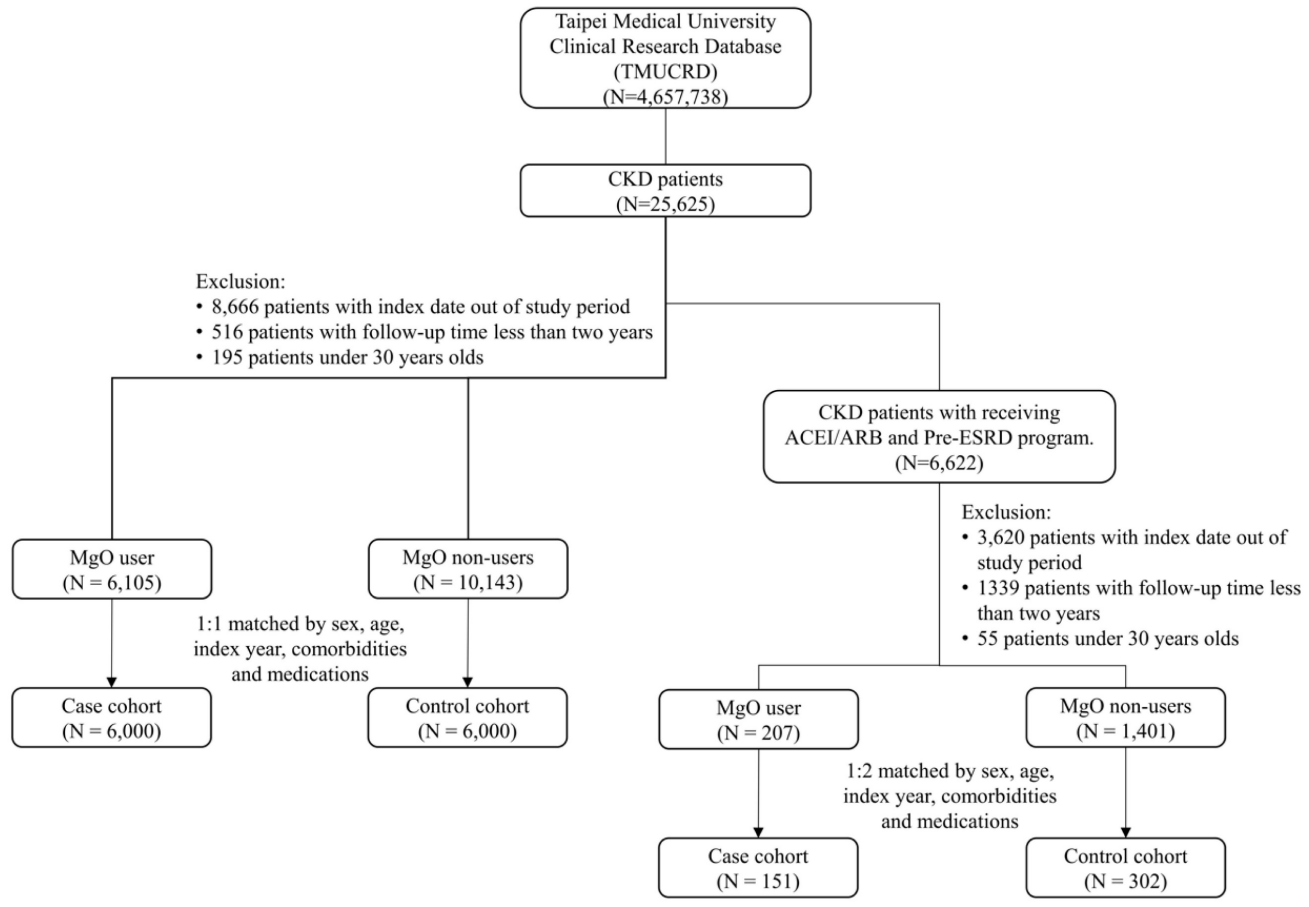
The study flow chart.

The patient selection process is shown in detail in Figure 1. Table 1a shows the baseline characteristics of all CKD patients' cohort. Before the matching, 6,105 MgO users and 10,143 MgO non-users were included. More male and younger patients were in the MgO group. The value of creatinine, eGFR and albumin in MgO users were lower than that of the MgO non-user. The distribution of comorbidities and medication between MgO users and non-users were different. After the propensity score matching, 6,000 MgO users and non-user were enrolled. Fifty percent for men and women. Most patients were in 60-70 years old. There were difference of creatinine and eGFR between two groups. Besides COPD, the proportions of comorbidities among two group of patients were similar. MgO users were more likely to take rivaroxaban, β-blocker, CCB, statin, metformin, DPP4 and insulin. About 73 % of MgO group with medication possession ratio (MPR) lower than 40%. Table 1b is the baseline characteristics of CKD patients receiving ACEI/ARB and Pre-ESRD program cohort. In the total of 1,608 patients, 1,401 was MgO non-user and 207 was MgO user. Male patients present more in MgO user than that in MgO non-user. Creatinine value of MgO user was higher. More patients with history of diabetes and hyperlipidemia and medication of statin, metformin and sulfonylureas in the control group than in the MgO group. But, MgO group had more patients with dementia. The matching cohort was consisting of 151 MgO users and 302 non-users. The mean of creatinine and albumin was lower in MgO user than the non-user. There were more patients with hypertension and congestive heart failure, but less patients with ischemic stroke in the control group. The percentage of MgO user with <40%, 40-80% and 80% were 77.48%, 12.58% and 9.93%, respectively.

### Clinical outcomes for CKD patients

**Figure 2:**
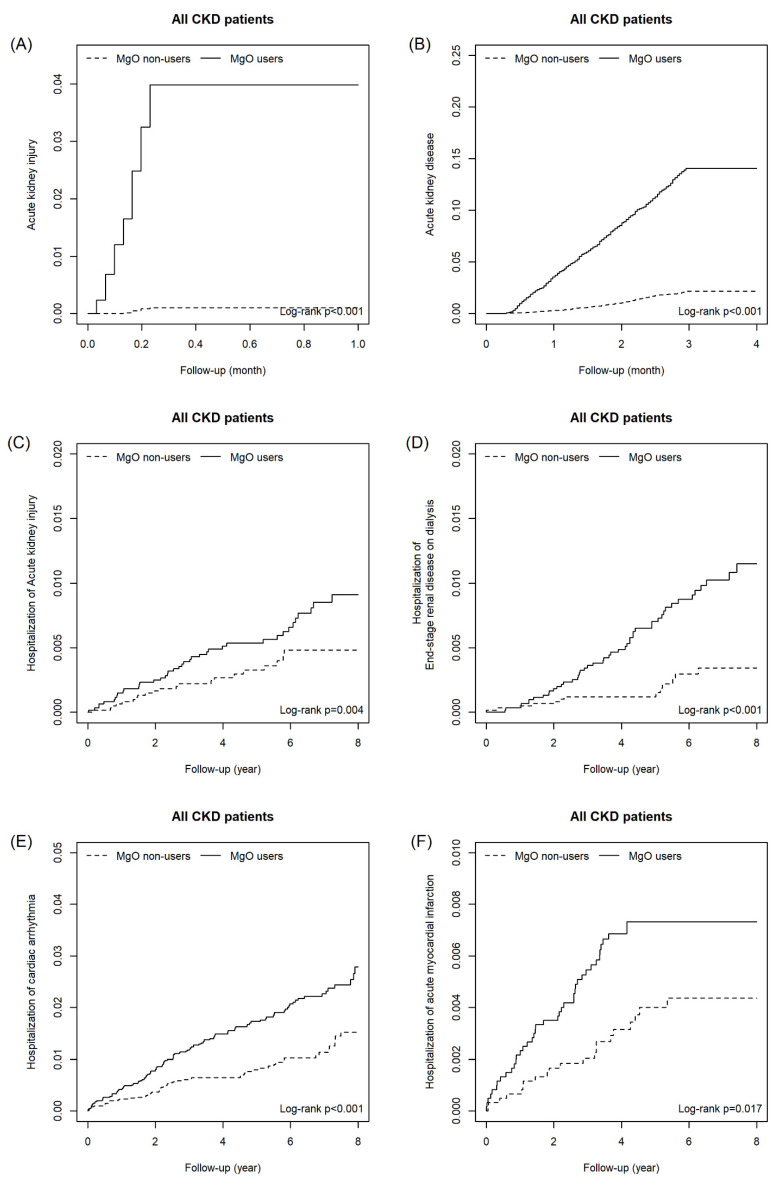
The cumulative incidence of Acute kidney injury (A), Acute kidney disease (B), Hospitalization of acute kidney injury (C), Hospitalization of end-stage renal disease on dialysis (D), Hospitalization of cardiac arrhythmia (E) and Hospitalization of acute myocardial infarction (F) in CKD patients.

**Figure 3:**
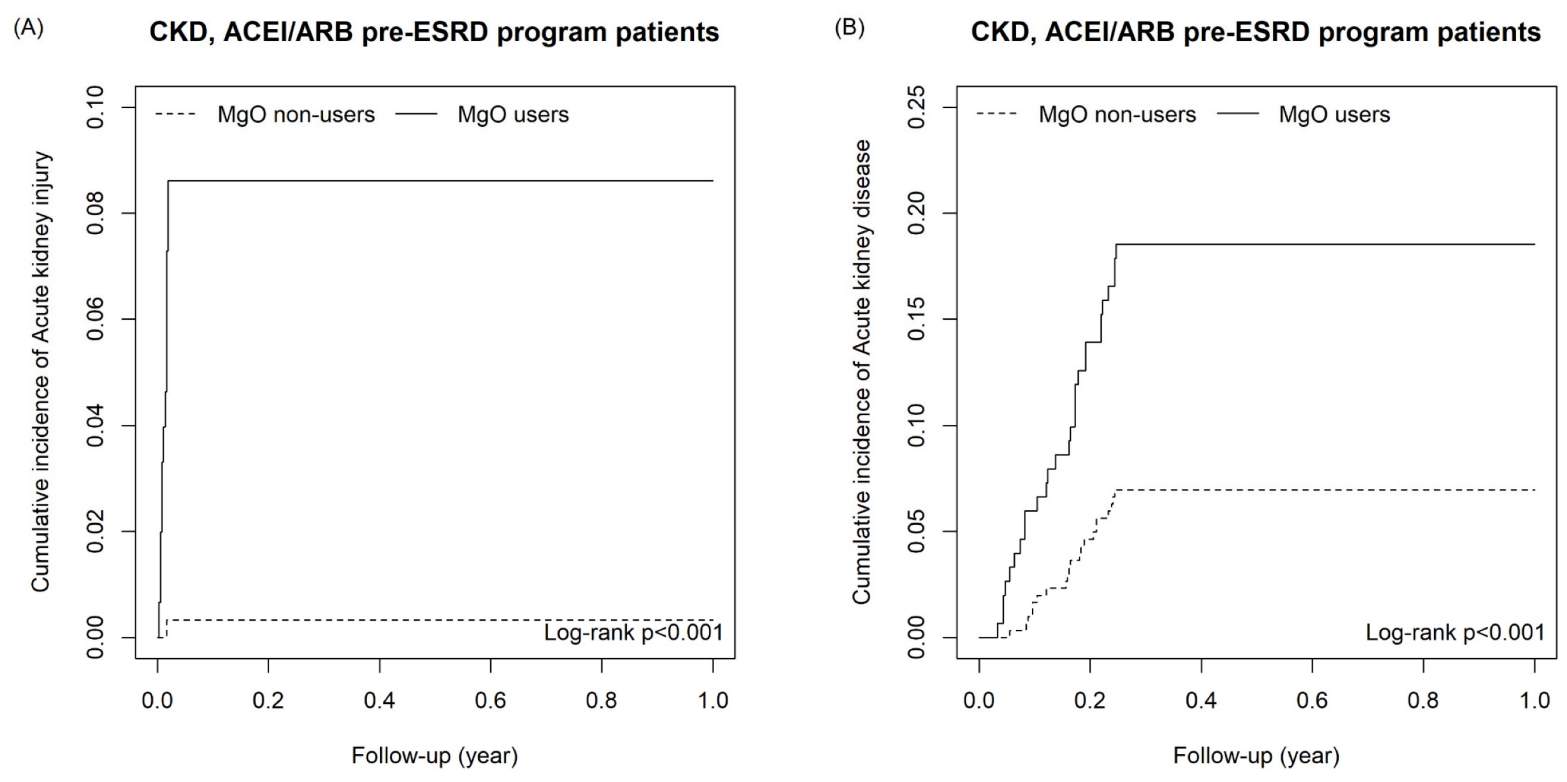
The cumulative incidence of Acute kidney injury (A) and Acute kidney disease (B) in CKD, ACEI/ARB pre-ESRD program patients.

As shown as Table 2a, the risk of outcomes was higher in MgO user relative to the control group in all CKD cohort with and without matching. For all CKD patients, the adjusted HR of AKI, AKD, hospitalization of acute kidney injury, end-stage renal disease on dialysis, congestive heart failure with acute pulmonary edema, cardiac arrhythmia and acute myocardial infarction were 45.40 (22.4, 92.07), 6.43(5.53, 7.47), 1.87(1.16, 2.99), 2.59(1.58, 4.22), 2.79(1.07,7.27), 2.18(1.66, 2.86) and 1.75(1.12, 2.73), respectively. For the matching cohort, AKI (aHR=37.00, 95%CI=16.46, 83.41), AKD (aHR=6.26, 95%CI=5.20, 7.53), hospitalization of acute kidney injury (aHR=1.97, 95%CI=1.15, 3.37), end-stage renal disease on dialysis (aHR=2.06, 95%CI=1.49, 2.84) and cardiac arrhythmia (aHR=1.86, 95%CI=1.10, 3.15) remained significantly higher. Figure 2 presents the cumulative incidence of six outcomes between MgO user and non-user in all CKD patients. Table 2b presents that in the un-matched cohort of CKD patients with receiving ACEI/ARB and Pre-ESRD program, the risk of all outcomes were higher in the MgO user than that in the MgO non-user. For the matched cohort, patients used MgO have a higher risk of AKI (aHR=16.1; 95%CI=1.90, 136.96) and AKD (aHR=2.79; 95%CI=1.50, 5.20). The cumulative incidence curves of AKI and AKD for CKD patients with receiving ACEI/ARB and Pre-ESRD program presented in Figure 3.

**Figure 4:**
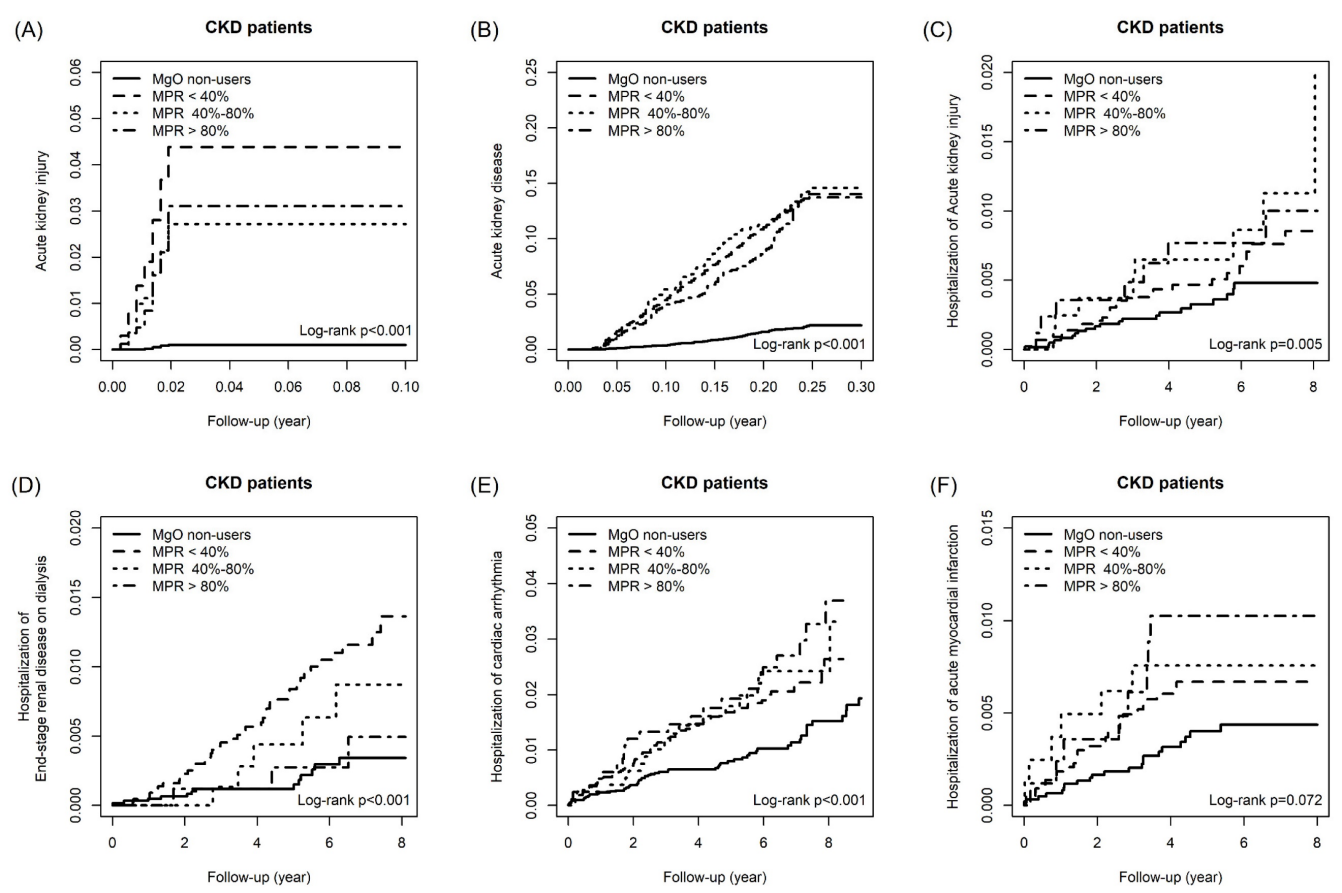
The cumulative incidence of Acute kidney injury (A), Acute kidney disease (B), Hospitalization of acute kidney injury (C), Hospitalization of end-stage renal disease on dialysis (D), Hospitalization of cardiac arrhythmia (E) and Hospitalization of acute myocardial infarction (F) in CKD patients among difference medication possession ratios.

Considering the association between medication possession ratio (MPR) and outcomes in all CKD patients, Table 3 illustrated that patients with MPR <40%, 40%-80% and >80% increase the risk of AKI by 41.10 folds (95%CI=18.23, 92.97), 25.50 folds (95%CI=10.35, 63.13) and 28.40 folds (95%CI =11.71, 69.30), respectively, compared to the MgO non-user. For the outcome of AKD, the aHR of MPR<40%, 40-80% and >80% were 6.21 (95%CI=5.13, 7.50), 6.83 (95%CI=1.50, 6.87) and 6.02 (95%CI=4.86, 7.74). Only patients with MPR<40% (aHR= 1.86; 95%CI=1.03, 3.34) and 40-80% (aHR= 3.21; 95%CI=1.50, 6.87) had a higher risk of hospitalization of acute kidney injury relative to the control group. After controlling the confounder, MPR <40% user increase the risk of hospitalization of end-stage renal disease on dialysis by 3.46 times (95%CI=1.80, 6.64). With MgO non-user as the reference group, the aHR of hospitalization of cardiac arrhythmia for MPR<40% patients were 1.96 (95%CI=1.39, 2.77), that for MPR 40-80% patients were 2.21 (95%CI=1.30, 3.78) and that for MPR>80% patients were 2.37 (95%CI=1.39, 2.77). Only patients with MPR >80% (aHR=2.66; 95%CI=1.17, 6.04) increase the risk of hospitalization of acute myocardial infarction. Figure 4 is the cumulative incidence curves of all outcomes among different MPR patients.

### Association of different CKD stages with outcomes

Table 4a and b present the associations between different CKD stages and clinical outcomes among MgO users, both in unmatched and propensity score-matched cohorts. For AKI, the risk rose progressively with advancing CKD stage. Compared with stage 1, patients in stage 3-5 exhibited significantly higher risks, with aHR of 2.16 for stage 3, 3.97 for stage 4, and 1.60-2.38 for stage 5 in unmatched and matched cohorts. For AKD, risk was strongly stage-dependent. Patients with stage 3 had a 1.70-fold higher risk, stage 4 a 2.60-fold risk, and stage 5 a 3.70-fold risk in the unmatched cohort, with similar magnitudes after matching. Notably, stage 5 patients demonstrated the highest incidence rates (197-229 per 1000 person-years). Regarding hospitalization for AKI, CKD stage 3-5 patients carried substantially elevated risks. Stage 4 was associated with aHR of 6.06-4.41, while stage 5 patients had 3.73-fold higher risks than stage 1 after matching. For progression to ESRD requiring dialysis, the risks increased steeply by CKD stage. Compared with stage 1, adjusted risks were 6.72-fold higher in stage 4 and 27.9-fold higher in stage 5 in the unmatched cohort; after matching, stage 5 remained markedly elevated (aHR 13.5, 95% CI 10.2-17.9). For cardiac arrhythmia, stage 3 and stage 4 patients consistently had higher risks, with aHR of ~2.0 and 3.2-4.2, respectively, while stage 5 patients also showed increased but more variable risks. For acute myocardial infarction, the associations were weaker; only stage 5 showed a modest increase in the unmatched analysis (aHR 3.24, 95% CI 0.96-10.93), though confidence intervals were wide. Overall, both analyses confirmed a clear dose-response relationship: advancing CKD stage in MgO users was associated with progressively higher risks of AKI, AKD, dialysis initiation, and arrhythmia, with the strongest effects observed in stage 4-5 disease.

## Discussion

To our knowledge, this is the first study to explore the associations between MgO use and the risk of cardiovascular events and dialysis in patients with advanced stage of CKD treated with ACEIs/ARBs. In this large cohort study, we examined the association between MgO use and clinical outcomes, with a focus on AKI, AKD, ESRD, and cardiovascular complications. These findings suggest that CKD patients, particularly those in more advanced stages of renal impairment, may be highly vulnerable to the adverse renal and cardiovascular outcomes associated with MgO use. Given the widespread use of MgO as a common therapeutic agent for constipation, dyspepsia, and suspected magnesium deficiency in clinical practice, these observations are clinically important. Physicians must carefully evaluate the balance between potential therapeutic benefits and the risks of harm when considering MgO therapy in this population. Close monitoring of renal function, cardiovascular status, and serum magnesium levels may be warranted to mitigate the likelihood of adverse outcomes. MgO use was associated with markedly increased risks; however, the magnitude of these associations suggests potential residual confounding or indication bias that cannot be fully excluded. Ultimately, further prospective studies are needed to clarify the causal relationship and to establish safer strategies for magnesium supplementation in CKD patients.

### Magnesium homeostasis in humans

Laboratory indices, including serum total magnesium and ionized Mg²⁺, are also imperfect markers. Their values are influenced by circadian rhythm, systemic inflammation, hypoalbuminemia, renal function, and concurrent medication use. Moreover, serum concentrations reflect only ~1% of total body magnesium, as the majority (~90%) is stored intracellularly within bone and muscle tissue 24-26. Mobilization from these stores can maintain serum magnesium within the normal range despite significant depletion. Consequently, normal serum magnesium levels may not accurately represent total body stores, and subclinical magnesium deficiency may remain undetected. Such hidden deficits can exert long-term physiological effects, increasing susceptibility to cardiovascular disease, metabolic disorders, and other chronic conditions 25-27.

### Clinical outcomes and MgO use

Our results revealed that MgO users had significantly higher adjusted risks of AKI, AKD, and progression to ESRD, both in unmatched and propensity score-matched cohorts. These findings appear paradoxical, as prior observational studies have suggested that higher serum magnesium is protective against vascular calcification, cardiovascular mortality, and sudden cardiac death in CKD. One possible explanation is that MgO users in our cohort may have had worse baseline clinical profiles, including higher comorbidity burdens, gastrointestinal symptoms, or medication exposures, leading clinicians to prescribe MgO. Indeed, Table 1a and b demonstrated that MgO users had lower eGFR, lower albumin, and greater prevalence of comorbidities such as COPD and dementia. Although propensity matching minimized these imbalances, residual confounding cannot be excluded. Furthermore, poor adherence emerged as a critical determinant of outcomes. Patients with MPR <40% had a 41-fold increased risk of AKI and six-fold increased risk of AKD compared with non-users, and their risk of dialysis initiation and arrhythmia was significantly higher. These findings suggest that inconsistent MgO use may exacerbate fluctuations in magnesium balance, exposing patients to both hypomagnesemia and treatment gaps. In contrast, CKD patients with high adherence (MPR ≥80%) failed to show clear protective benefits, suggesting that MgO supplementation may be inadequate to offset the numerous risk factors in CKD and could potentially contribute to adverse outcomes.

### Dysmagnesemia and cardiovascular complications in CKD

The association between dysmagnesemia and cardiovascular disease has been reported. Low magnesium levels are linked to hypertension, insulin resistance, left ventricular hypertrophy, and arrhythmogenesis. Our study adds to this evidence by showing that MgO users, particularly those with low adherence, faced higher risks of cardiac arrhythmia and myocardial infarction. Interestingly, only patients with MPR ≥80% demonstrated a significantly increased risk of myocardial infarction, suggesting that both magnesium status and potential adverse effects of MgO therapy warrant further investigation. One possible hypothesis is that MgO-induced gastrointestinal side effects or other unknown cardiovascular harmfulness, such as diarrhea, may worsen volume status and precipitate cardiovascular stress in CKD patients. Previous research results regarding magnesium supplementation in CKD were controversial. Higher serum magnesium was inversely associated with cardiovascular mortality in hemodialysis patients 8. Other epidemiological studies emphasized the protective role of magnesium against vascular calcification 9, 14. However, several studies highlighted the unanswered questions regarding the safety of magnesium supplementation in patients with advanced stages of CKD, particularly in the context of limited renal excretion 28-33.

Alternative strategies to optimize magnesium status—such as dietary counseling, tailored dialysate magnesium concentration, or use of other magnesium formulations with better bioavailability—should be considered in CKD care. Clinicians should remain alert to the potential adverse effects of MgO, such as gastrointestinal intolerance and the risk of hypermagnesemia in patients with advanced CKD, and adjust therapy as appropriate. Several studies also highlighted important safety considerations regarding the widespread use of MgO for constipation, particularly in populations with impaired renal function. Terashima *et al.* reported that MgO administration was associated with an increased risk of hospital readmission among patients with both heart failure and constipation 34. This finding suggests that even standard-dose MgO may contribute to clinical instability in vulnerable cardiovascular populations, potentially through fluid-electrolyte disturbances, altered neurohormonal responses, or subclinical accumulation of magnesium in the setting of reduced renal perfusion. Ishii* et al.* further demonstrated that low creatinine clearance and higher daily magnesium intake were significant and independent predictors of hypermagnesemia in older adults 35. Because creatinine clearance declines physiologically with age—and much more profoundly in patients with CKD—these results underscore the heightened susceptibility of CKD patients to magnesium accumulation even when MgO is prescribed at conventional doses. Hypermagnesemia in advanced CKD can develop insidiously and may remain asymptomatic until serum magnesium concentrations exceed the compensatory threshold.

Mori *et al.* also presented the clinical manifestations of MgO-induced hypermagnesemia in constipated patients 36. Reported symptoms ranged from nonspecific findings such as nausea, lethargy, and hypotonia to more serious consequences including bradyarrhythmia, hypotension, and depressed consciousness. Notably, many affected individuals were elderly and had unrecognized renal impairment, supporting the concept that routine monitoring is essential when MgO is used in populations at risk. Collectively, these studies indicate that CKD patients—particularly those with stage 3-5 disease, heart failure comorbidity, or older age—are at substantial risk for magnesium accumulation and adverse events 36-38. Given the impaired renal excretion of magnesium and the narrow safety margin in CKD, clinicians should consider alternative constipation therapies, closely monitor serum magnesium levels, and individualize dosing strategies when MgO is deemed necessary.

In a large CKD cohort study, serum magnesium levels <1.9 mg/dL or >2.1 mg/dL were linked to increased all-cause mortality. Low magnesium was reported to be associated with new-onset atrial fibrillation but not with composite cardiovascular events 39. Further research is warranted to define the optimal serum magnesium range in CKD for preventing adverse outcomes. Our study findings extend these observations by demonstrating that real-world MgO prescriptions do not necessarily translate into improved outcomes. Additionally, an evidence-based serum magnesium reference interval that reflects optimal health, current dietary patterns, and population characteristics is urgently needed. In patients with CKD, where magnesium homeostasis is frequently disrupted, such a reference range would be particularly valuable for guiding clinical decision-making and preventing both deficiency- and excess-related complications. Patients with CKD commonly exhibit cardiovascular-kidney-metabolic (CKM) syndrome. A magnesium-depleted state, defined as a magnesium depletion score (MgDS) ≥ 2, represents a significant risk factor for progression to advanced CKM stages. This finding shows that early identification of magnesium deficiency and optimization of magnesium nutritional status may help reduce the risk of advancing CKM 40.

### Strengths and limitations

The strengths of this study include its large sample size, long follow-up, and detailed adjustment for comorbidities and medications. The use of MPR provided an objective measure of adherence, which revealed strong associations with outcomes. This study has several implications for clinical practice. First, routine monitoring of serum magnesium levels is essential, as normal values may mask subclinical magnesium deficiency due to mobilization from bone and muscle stores. Second, adherence to MgO therapy should be carefully assessed, as poor compliance markedly increased the risks of both renal and cardiovascular events. Several limitations must be acknowledged. In this cohort study, serum magnesium levels were not available for all patients and may not accurately reflect total body magnesium stores. Residual confounding by indication is possible, as patients prescribed MgO may have had unmeasured differences in nutritional status or gastrointestinal conditions. Several outcomes, particularly AKI and AKD, demonstrate high adjusted hazard ratios. While statistically significant, these estimates may raise concerns regarding residual confounding or outcome misclassification. Baseline renal vulnerability, surveillance bias, or medication indication bias may have impacts on the clinical outcomes. Although the propensity score matching was rigorously performed, the potential for residual confounding cannot be completely excluded. Patients receiving MgO therapy may have unmeasured characteristics—such as underlying gastrointestinal disorders, frailty, or nutritional deficiencies—that are not fully captured in administrative data and could independently influence renal or cardiovascular outcomes. In fact, serum magnesium reflects only a small fraction of total body magnesium and may not accurately represent intracellular or tissue magnesium stores. This clarification strengthens the biological interpretation of our findings and prevents misinterpretation of MgO as a direct causal agent. Additionally, the observational design precludes causal inference, and further interventional trials are warranted to clarify the role of magnesium supplementation in CKD patients.

## Conclusion

In summary, while MgO remains a commonly used laxative in East Asia, its use in CKD requires a risk-stratified approach. Patients with mild CKD and without cardiovascular comorbidities or frailty may still use MgO cautiously at low doses with periodic monitoring of serum magnesium. However, individuals with moderate-to-severe CKD, older adults with reduced creatinine clearance, and those with heart failure or other high-risk features face a substantially higher likelihood of magnesium accumulation and related renal or cardiovascular complications. Integrating the current results with existing evidence underscores the importance of prioritizing safer alternatives, such as polyethylene glycol or lactulose, in these vulnerable groups. Clinicians should remain alert for early manifestations of hypermagnesemia and tailor constipation therapy to each patient's renal function and comorbidity profile to optimize safety in CKD management.

## Figures and Tables

**Table 1a:** Baseline characteristics of CKD patients.

	Without matching					1:1 PS matching				
	Control		Magnesium oxide			Control		Magnesium oxide		
	N=10143		N=6105			N=6000		N=6000		
Variables	n	%	n	%	p-value	n	%	n	%	p-value
Sex					0.010					0.559
Female	4809	47.41	3022	49.50		3005	50.08	2973	49.55	
Male	5334	52.59	3083	50.50		2995	49.92	3027	50.45	
Age, year					<0.001					0.006
30-70	5954	58.70	3108	50.91		3213	53.55	3062	51.03	
>70	4189	41.30	2997	49.09		2787	46.45	2938	48.97	
Mean (SD)	66.74	(14.22)	68.85	(13.29)	<0.001	68.74	(13.77)	68.8	(13.27)	0.796
Serum data, mean (SD)										
Mg (mg/Dl)	2.21	(0.42)	2.08	(0.43)	0.005	2.23	(0.39)	2.08	(0.44)	0.004
Creatinine (mg/dL)	1.47	(1.92)	1.31	(1.43)	<0.001	1.18	(1.27)	1.11	(1.04)	0.018
eGFR (mL/min/1.73m2)	77.52	(43.74)	74.14	(48.97)	0.011	69.88	(34.31)	64.16	(32.36)	<0.001
Hemoglobin (g/dL)	10.81	(1.83)	10.87	(1.94)	0.874	10.99	(1.84)	10.91	(1.94)	0.852
Albumin (g/dL)	3.71	(0.62)	3.52	(0.60)	0.016	3.68	(0.67)	3.53	(0.61)	0.160
Comorbidities										
Hypertension	4590	45.25	2907	47.62	0.003	2847	47.45	2854	47.57	0.898
Diabetes	5141	50.69	2949	48.30	0.003	2865	47.75	2897	48.28	0.559
Hyperlipidemia	2894	28.53	1895	31.04	0.001	1874	31.23	1865	31.08	0.859
IHD	3470	34.21	1713	28.06	<0.001	1652	27.53	1685	28.08	0.501
Ischemic stroke	125	1.23	153	2.51	<0.001	112	1.87	143	2.38	0.050
Congestive heart failure	1049	10.34	509	8.34	<0.001	505	8.42	502	8.37	0.921
AF	129	1.27	113	1.85	0.003	100	1.67	111	1.85	0.445
PAD	219	2.16	103	1.69	0.037	101	1.68	99	1.65	0.887
COPD	880	8.68	850	13.92	<0.001	730	12.17	818	13.63	0.017
CLD	879	8.67	666	10.91	<0.001	646	10.77	652	10.87	0.860
Dementia	381	3.76	350	5.73	<0.001	307	5.12	337	5.62	0.224
Medication										
Antiplatelet	4859	47.91	2946	48.26	0.665	2836	47.27	2888	48.13	0.342
Warfarin	379	3.74	215	3.52	0.480	216	3.60	214	3.57	0.922
Rivaroxaban	33	0.33	51	0.84	<0.001	28	0.47	50	0.83	0.012
ACEI/ARB	4159	41.00	2660	43.57	0.001	2525	42.08	2609	43.48	0.121
Beta-2 blocker	3902	38.47	2524	41.34	<0.001	2306	38.43	2474	41.23	0.002
CCB	3857	38.03	2801	45.88	<0.001	2371	39.52	2731	45.52	<0.001
Statin	3208	31.63	2109	34.55	<0.001	1936	32.27	2074	34.57	0.008
Metformin	3253	32.07	2268	37.15	<0.001	1929	32.15	2225	37.08	0.000
Thiazolidinedione	648	6.39	371	6.08	0.427	378	6.30	367	6.12	0.677
Sulfonylureas	2788	27.49	1766	28.93	0.048	1667	27.78	1729	28.82	0.209
AGIs	1074	10.59	681	11.15	0.260	637	10.62	669	11.15	0.348
DPP4	1315	12.96	1090	17.85	<0.001	811	13.52	1066	17.77	<0.001
Insulin	2356	23.23	2201	36.05	<0.001	1448	24.13	2146	35.77	<0.001
PPI	1474	14.53	1393	22.82	<0.001	951	15.85	1393	22.82	<0.001
Thiazide	875	8.63	640	10.48	<0.001	544	9.07	640	10.48	0.0137
Loop diuretic	1730	17.06	1339	21.93	<0.001	1070	17.83	1339	21.93	<0.001
MPR										
<40%			4426	72.50				4354	72.57	
40-80%			827	13.55				809	13.48	
>80%			852	13.96				837	13.95	
CKD stage					<0.001					<0.001
1	521	5.14	549	8.99		324	5.40	527	8.78	
2	587	5.79	934	15.3		403	6.72	912	15.2	
3	374	3.69	992	16.25		260	4.33	965	16.08	
4	128	1.26	243	3.98		91	1.52	240	4.00	
5	8533	84.13	3387	55.48		4922	82.03	3356	55.93	

IHD: ischemic heart disease; AF: Atrial fibrillation; PAD: peripheral artery disease; COPD: chronic obstructive pulmonary disease; CLD: chronic liver disease; ACEI: angiotensin-converting enzyme inhibitor; ARB: angiotensin II receptor blocker; CCB: calcium channel blockers; AGIs: alpha-glucosidase inhibitors; DPP4: dipeptidyl peptidase 4; PPI: proton-pump inhibitor; MPR: medication possession ratio.

**Table 1b:** Baseline characteristics of study for CKD patients with receiving ACEI/ARB and Pre-ESRD program.

	Without matching						1:1 PS matching				
	Control			Magnesium oxide			Control		Magnesium oxide		
	N=1401			N=207			N=302		N=151		
Variables	n	%		n	%	p-value	n	%	n	%	p-value
Sex						0.007					0.842
Female	742	52.96		89	43.00		161	53.31	79	52.32	
Male	659	47.04		118	57.01		141	46.69	72	47.68	
Age						0.521					0.466
30-80	985	70.31		141	68.12		265	87.75	136	90.07	
>80	416	29.69		66	31.88		37	12.25	15	9.93	
Mean (SD)	72.5	(11.83)		73.05	(11.97)	0.532	66.37	(11.45)	64.97	(11.81)	0.224
Comorbidities											
Hypertension	1162	82.94		170	82.13	0.772	246	81.46	109	72.19	0.024
Diabetes	1089	77.73		135	65.22	<0.001	237	78.48	118	78.15	0.936
Hyperlipidemia	694	49.54		75	36.23	<0.001	148	49.01	60	39.74	0.062
IHD	744	53.11		98	47.34	0.121	148	49.01	70	46.36	0.595
Ischemic stroke	54	3.85		6	2.90	0.498	6	1.99	14	9.27	<0.001
Congestive heart failure	256	18.27		42	20.29	0.486	63	20.86	15	9.93	0.004
AF	68	4.85		6	2.90	0.210	8	2.65	2	1.33	0.366
PAD	51	3.64		6	2.90	0.590	7	2.318	5	3.311	0.535
COPD	240	17.13		41	19.81	0.344	44	14.57	27	17.88	0.361
CLD	153	10.92		29	14.01	0.190	45	14.90	14	9.27	0.093
Dementia	124	8.85		31	14.98	0.005	10	3.31	12	7.95	0.030
Medication											
Antiplatelet	1140	81.37		158	76.33	0.086	233	77.15	106	70.20	0.108
Warfarin	1140	81.37		158	76.33	0.086	233	77.15	106	70.20	0.108
Rivaroxaban	43	3.07		4	1.93	0.365	8	2.65	6	3.97	0.443
ACEI/ARB	1387	100.00		191	100.00		300	100.00	142	100.00	
Beta-2 blocker	1032	73.66		140	67.63	0.069	228	75.50	107	70.86	0.289
CCB	1136	81.09		176	85.02	0.172	234	77.48	116	76.82	0.874
Statin	940	67.10		115	55.56	0.001	194	64.24	89	58.94	0.272
Metformin	874	62.38		86	41.55	<0.001	189	62.58	104	68.87	0.187
Thiazolidinedione	180	12.85		19	9.18	0.135	38	12.58	14	9.27	0.297
Sulfonylureas	743	53.03		86	41.55	0.002	153	50.66	77	50.99	0.947
AGIs	327	23.34		55	26.57	0.308	56	18.543	26	17.22	0.730
DPP4	327	23.34		55	26.57	0.308	56	18.543	26	17.22	0.730
Insulin	327	23.34		55	26.57	0.308	56	18.543	26	17.22	0.730
PPI	440	31.41		90	43.48	0.001	99	32.78	61	40.40	0.110
Thiazide	362	25.84		62	29.95	0.210	66	21.85	35	23.18	0.750
Loop diuretic	616	43.97		137	66.18	<0.001	137	45.36	68	45.03	0.947
MPR											
<40%				149	71.98				117	77.48	
40-80%				25	12.08				19	12.58	
>80%				33	15.94				15	9.93	
CKD stage						<0.001					<0.001
1	14	1.00		0	0.00		6	1.99	1	0.66	
2	115	8.21		4	1.93		38	12.58	12	7.95	
3	731	52.18		85	41.06		128	42.38	103	68.21	
4	129	9.21		52	25.12		27	8.94	17	11.26	
5	412	29.41		66	31.88		103	34.11	18	11.92	

IHD: ischemic heart disease; AF: Atrial fibrillation; PAD: peripheral artery disease; COPD: chronic obstructive pulmonary disease; CLD: chronic liver disease; ACEI: angiotensin-converting enzyme inhibitor; ARB: angiotensin II receptor blocker; CCB: calcium channel blockers; AGIs: alpha-glucosidase inhibitors; DPP4: dipeptidyl peptidase 4; PPI: proton-pump inhibitor; MPR: medication possession ratio.

**Table 2a:** Risk outcomes for CKD patients.

	Control			Magnesium oxide						
Outcome	n	PY	IR	n	PY	IR	cHR	(95% CI)	aHR^ꝉ^	(95% CI)
*Without matching*										
Acute kidney injury	8	62316	0.13	250	33107	7.55	52.8	(26.13, 106.81)***	45.4	(22.4, 92.07)***
Acute kidney disease	221	61140	3.61	892	29694	30.04	7.20	(6.21, 8.34)***	6.43	(5.53, 7.47)***
Hospitalization of acute kidney injury	37	62252	0.59	45	34354	1.31	2.41	(1.52, 3.80)***	1.87	(1.16, 2.99)**
Hospitalization of end-stage renal disease on dialysis	33	62314	0.53	54	34354	1.57	3.12	(1.94, 5.02)***	2.59	(1.58, 4.22)***
Hospitalization of congestive heart failure with acute pulmonary edema	8	62345	0.13	13	34436	0.38	3.37	(1.33, 8.53)*	2.79	(1.07, 7.27)*
Hospitalization of cardiac arrhythmia	192	61411	3.13	197	33639	5.86	2.41	(1.86, 3.14)***	2.18	(1.66, 2.86)***
Hospitalization of acute myocardial infarction	40	62188	0.64	41	34289	1.20	1.71	(1.10, 2.64)*	1.75	(1.12, 2.73)*
Death	471	72022	6.54	579	39934	14.5	2.11	(1.87, 2.39)***	1.78	(1.56, 2.02)***
*1:1 PS matching*										
Acute kidney injury	6	33713	0.18	239	32600	7.33	40.4	(18.01, 91.02)***	37.0	(16.46, 83.41)***
Acute kidney disease	132	33038	4.00	844	29366	28.74	6.82	(5.68, 8.20)***	6.26	(5.20, 7.53)***
Hospitalization of acute kidney injury	23	33676	0.68	44	33787	1.3	2.14	(1.26, 3.64)**	1.97	(1.15, 3.37)*
Hospitalization of end-stage renal disease on dialysis	16	33713	0.47	53	33784	1.57	3.70	(1.97, 6.96)***	3.13	(1.65, 5.94)***
Hospitalization of congestive heart failure with acute pulmonary edema	5	33739	0.15	12	33865	0.35	2.31	(0.81, 6.59)	1.94	(0.67, 5.63)
Hospitalization of cardiac arrhythmia	127	33081	3.84	180	33121	5.43	2.08	(1.52, 2.86)***	2.06	(1.49, 2.84)***
Hospitalization of acute myocardial infarction	21	33662	0.62	41	33719	1.22	1.87	(1.11, 3.17)*	1.86	(1.10, 3.15)*
Death	252	39488	6.38	563	39277	14.33	2.15	(1.85, 2.49)***	2.10	(1.80, 2.44)***

N: number of event; PY: person-year; IR: incidence rate per 1000 person-years; cHR: crude hazard ratio; aHR: adjusted hazard ratio; ^ꝉ^: adjusted for sex, age, comorbidities and medication, *: p-value<0.05; **: p-value<0.01; ***: p-value<0.001.

**Table 2b:** Risk outcomes for CKD patients with receiving ACEI/ARB.

	Control			Magnesium oxide						
Outcome	n	PY	IR	n	PY	IR	cHR	(95% CI)	aHR	(95% CI)
*Without matching*										
Acute kidney injury	3	5318	0.56	13	805	16.15	29.9	(8.54, 105.21)***	47.0	(10.7, 206.68)***
Acute kidney disease	120	4934	24.32	62	635	97.56	4.02	(2.96, 5.47)***	3.74	(2.71, 5.17)***
Hospitalization of acute kidney injury	12	5315	2.26	8	840	9.52	4.17	(1.7, 10.23)**	6.34	(2.34, 17.19)***
Hospitalization of end-stage renal disease on dialysis	18	5305	3.39	14	833	16.81	4.15	(1.83, 9.38)***	4.08	(1.61, 10.35)**
Hospitalization of congestive heart failure with acute pulmonary edema	2	5329	0.38	1	852	1.17	3.19	(0.29, 35.17)	NA	-
Hospitalization of cardiac arrhythmia	23	5237	4.39	8	829	9.65	2.70	(0.95, 7.66)	3.59	(1.14, 11.31)*
Hospitalization of acute myocardial infarction	1	5326	0.19	3	847	3.54	20.3	(2.12, 195.8)**	26.9	(2.39, 304.42)**
Death	284	6390	44.44	93	957	97.15	2.23	(1.77, 2.83)***	2.04	(1.58, 2.65)***
*1:2 PS matching*										
Acute kidney injury	1	909	1.1	13	426	30.55	26.9	(3.53, 206.16)**	16.1	(1.90, 136.96)*
Acute kidney disease	21	847	24.79	28	385	72.66	2.86	(1.62, 5.03)***	2.79	(1.50, 5.20)**
Hospitalization of acute kidney injury	1	911	1.1	1	471	2.12	1.94	(0.12, 31.08)	NA	-
Hospitalization of end-stage renal disease on dialysis	9	899	10.02	3	467	6.42	0.61	(0.16, 2.24)	1.15	(0.19, 6.92)
Hospitalization of congestive heart failure with acute pulmonary edema	0	912	0	0	471	0	NA	-	NA	-
Hospitalization of cardiac arrhythmia	1	912	1.1	3	464	6.47	5.65	(0.59, 54.35)	NA	-
Hospitalization of acute myocardial infarction	1	912	1.1	1	468	2.14	NA	-	NA	-
Death	18	1189	15.14	6	614	9.78	0.64	(0.25, 1.62)	0.39	(0.13, 1.23)

N: number of events; PY: person-year; IR: incidence rate per 1000 person-years; cHR: crude hazard ratio; aHR: adjusted hazard ratio; ^ꝉ^: adjusted for sex, age, comorbidities and medication, *: p-value<0.05; **: p-value<0.01; ***: p-value<0.001.

**Table 3:** The association of MPR and outcomes in the matched cohort of CKD patients.

Outcome	n	PY	IR	cHR	(95% CI)	aHR	(95% CI)
*Acute kidney injury*							
Control	6	33713	0.18	1.00	(reference)	1.00	(reference)
MPR < 40%	191	22905	8.34	44.70	(19.84, 100.75)***	41.10	(18.23, 92.97)***
MPR 40%-80%	22	4652	4.73	27.40	(11.14, 67.77)***	25.50	(10.35, 63.13)***
MPR > 80%	26	5043	5.16	31.30	(12.91, 76.2)***	28.40	(11.71, 69.30)***
Acute kidney disease							
Control	132	33038	4.00	1.00	(reference)	1.00	(reference)
MPR < 40%	611	20709	29.50	6.81	(5.65, 8.22)***	6.21	(5.13, 7.50)***
MPR 40%-80%	118	4117	28.66	7.12	(5.55, 9.13)***	6.83	(5.32, 8.76)***
MPR > 80%	115	4540	25.33	6.59	(5.13, 8.46)***	6.02	(4.68, 7.74)***
Hospitalization of acute kidney injury							
Control	23	33676	0.68	1.00	(reference)	1.00	(reference)
MPR < 40%	26	23850	1.09	1.80	(1.00, 3.23)*	1.86	(1.03, 3.34)*
MPR 40%-80%	10	4758	2.10	3.43	(1.60, 7.34)**	3.21	(1.50, 6.87)**
MPR > 80%	8	5180	1.54	2.48	(1.09, 5.65)*	2.14	(0.94, 4.89)
Hospitalization of end-stage renal disease on dialysis							
Control	16	33713	0.47	1.00	(reference)	1.00	(reference)
MPR < 40%	45	23823	1.89	4.39	(2.31, 8.36)***	3.46	(1.80, 6.64)***
MPR 40%-80%	5	4763	1.05	2.67	(0.94, 7.58)	2.78	(0.97, 7.98)
MPR > 80%	3	5198	0.58	1.47	(0.42, 5.22)	1.54	(0.43, 5.52)
Hospitalization of congestive heart failure with acute pulmonary edema							
Control	5	33739	0.15	1.00	(reference)	1.00	(reference)
MPR < 40%	9	23888	0.38	2.53	(0.84, 7.62)	2.30	(0.75, 7.09)
MPR 40%-80%	2	4771	0.42	2.66	(0.52, 13.74)	1.71	(0.31, 9.42)
MPR > 80%	1	5206	0.19	1.15	(0.13, 9.88)	0.91	(0.10, 7.96)
Hospitalization of cardiac arrhythmia							
Control	127	33081	3.84	1.00	(reference)	1.00	(reference)
MPR < 40%	124	23404	5.30	1.98	(1.41, 2.78)***	1.96	(1.39, 2.77)***
MPR 40%-80%	27	4656	5.80	2.19	(1.29, 3.72)**	2.21	(1.30, 3.78)**
MPR > 80%	29	5061	5.73	2.46	(1.50, 4.04)***	2.37	(1.43, 3.92)***
Hospitalization of acute myocardial infarction							
Control	21	33662	0.62	1.00	(reference)	1.00	(reference)
MPR < 40%	27	23797	1.13	1.71	(0.97, 3.03)	1.69	(0.96, 3.00)
MPR 40%-80%	6	4745	1.26	2.01	(0.81, 4.99)	2.07	(0.84, 5.15)
MPR > 80%	8	5176	1.55	2.56	(1.13, 5.78)*	2.66	(1.17, 6.04)*

N: number of event; PY: person-year; IR: incidence rate per 1000 person-years; cHR: crude hazard ratio; aHR: adjusted hazard ratio; MPR: Medication Possession Ratio; ^ꝉ^: adjusted for sex, age, comorbidities and medication, *: p-value<0.05; **: p-value<0.01; ***: p-value<0.001.

**Table 4a:** Association of different CKD stages with outcomes in the unmatched Cohort of MgO users.

	Without matching							1:1 PS matching						
Outcome	n	PY	IR	cHR	(95% CI)	aHR	(95% CI)	n	PY	IR	cHR	(95% CI)	aHR	(95% CI)
Acute kidney injury														
CKD stage 1	113	23066	4.90	1.00	(reference)	1.00	(reference)	118	22853	5.16	1.00	(reference)	1.00	(reference)
CKD stage 2	33	4695	7.03	1.16	(0.78, 1.70)	1.12	(0.76, 1.66)	24	3914	6.13	0.87	(0.56, 1.36)	0.90	(0.58, 1.40)
CKD stage 3	66	4048	16.31	2.79	(2.06, 3.78)***	2.16	(1.57, 2.98)***	54	4262	12.67	1.96	(1.42, 2.71)***	1.50	(1.07, 2.10)*
CKD stage 4	27	722	37.39	5.82	(3.82, 8.85)***	3.97	(2.55, 6.16)***	22	920	23.92	3.33	(2.11, 5.25)***	1.89	(1.17, 3.05)**
CKD stage 5	11	577	19.05	2.99	(1.61, 5.55)***	1.60	(0.84, 3.06)	21	651	32.27	4.41	(2.77, 7.01)***	2.38	(1.44, 3.93)***
Acute kidney disease														
CKD stage 1	449	21118	21.26	1.00	(reference)	1.00	(reference)	449	20862	21.52	1.00	(reference)	1.00	(reference)
CKD stage 2	138	4179	33.02	1.23	(1.01, 1.48)*	1.21	(1.00, 1.47)*	111	3548	31.28	1.06	(0.86, 1.31)	1.08	(0.87, 1.33)
CKD stage 3	178	3505	50.79	1.95	(1.64, 2.33)***	1.70	(1.41, 2.04)***	150	3770	39.79	1.45	(1.21, 1.75)***	1.20	(0.99, 1.46)
CKD stage 4	57	585	97.40	3.27	(2.48, 4.31)***	2.60	(1.96, 3.46)***	55	785	70.04	2.27	(1.72, 3.00)***	1.60	(1.19, 2.14)**
CKD stage 5	70	306	228.51	5.71	(4.44, 7.34)***	3.70	(2.82, 4.87)***	79	401	197.18	5.10	(4.02, 6.48)***	3.11	(2.39, 4.05)***
Hospitalization of acute kidney injury														
CKD stage 1	16	23682	0.68	1.00	(reference)	1.00	(reference)	23	23523	0.98	1.00	(reference)	1.00	(reference)
CKD stage 2	5	4846	1.03	1.55	(0.56, 4.28)	1.46	(0.52, 4.07)	3	4021	0.75	0.75	(0.22, 2.59)	0.79	(0.23, 2.72)
CKD stage 3	18	4349	4.14	5.62	(2.82, 11.23)***	4.23	(2.00, 8.93)***	8	4471	1.79	2.05	(0.90, 4.64)	1.51	(0.64, 3.54)
CKD stage 4	5	846	5.91	9.14	(3.33, 25.09)***	6.06	(2.06, 17.84)**	6	1027	5.84	6.68	(2.68, 16.62)***	4.41	(1.63, 11.93)**
CKD stage 5	1	632	1.58	2.45	(0.32, 18.54)	1.47	(0.18, 11.73)	4	745	5.37	6.20	(2.12, 18.13)***	3.73	(1.16, 12.04)*
Hospitalization of end-stage renal disease on dialysis														
CKD stage 1	196	23012	8.52	1.00	(reference)	1.00	(reference)	211	22801	9.25	1.00	(reference)	1.00	(reference)
CKD stage 2	24	4796	5.00	0.57	(0.37, 0.87)**	0.58	(0.38, 0.88)*	21	3986	5.27	0.53	(0.34, 0.84)**	0.56	(0.36, 0.88)*
CKD stage 3	61	4256	14.33	1.64	(1.23, 2.19)***	1.40	(1.03, 1.89)*	43	4414	9.74	1.01	(0.73, 1.40)	0.84	(0.59, 1.17)
CKD stage 4	62	729	85.06	9.40	(7.05, 12.53)***	6.72	(4.94, 9.13)***	56	913	61.34	6.07	(4.51, 8.17)***	3.88	(2.81, 5.34)***
CKD stage 5	106	207	510.97	44.50	(34.84, 56.86)***	27.90	(21.09, 37.12)***	100	356	281.04	24.10	(18.90, 30.74)***	13.50	(10.17, 17.92)***
Hospitalization of congestive heart failure with acute pulmonary edema														
CKD stage 1	9	23695	0.38	1.00	(reference)	1.00	(reference)	8	23538	0.34	1.00	(reference)	1.00	(reference)
CKD stage 2	1	4851	0.21	0.72	(0.09, 5.72)	0.59	(0.07, 4.82)	1	4023	0.25	1.02	(0.13, 8.30)	0.91	(0.11, 7.79)
CKD stage 3	3	4389	0.68	2.06	(0.56, 7.63)	1.72	(0.42, 7.03)	1	4497	0.22	0.81	(0.10, 6.50)	0.51	(0.06, 4.76)
CKD stage 4	0	865	0.00					2	1043	1.92	7.42	(1.56, 35.41)*	6.48	(1.12, 37.53)*
CKD stage 5	0	637	0.00					0	763	0.00	NA	-	NA	-
Hospitalization of cardiac arrhythmia														
CKD stage 1	105	23302	4.51	1.00	(reference)	1.00	(reference)	94	23139	4.06	1.00	(reference)	1.00	(reference)
CKD stage 2	27	4759	5.67	1.42	(0.87, 2.31)	1.42	(0.87, 2.33)	23	3955	5.82	1.44	(0.84, 2.46)	1.50	(0.87, 2.57)
CKD stage 3	45	4168	10.80	2.23	(1.44, 3.44)***	2.01	(1.26, 3.20)**	41	4324	9.48	1.79	(1.11, 2.88)*	1.71	(1.03, 2.83)*
CKD stage 4	13	796	16.34	3.99	(2.05, 7.75)***	4.16	(2.08, 8.29)***	15	968	15.49	3.40	(1.74, 6.62)***	3.23	(1.59, 6.53)**
CKD stage 5	7	614	11.40	2.61	(1.05, 6.46)*	2.39	(0.93, 6.14)	7	735	9.53	2.27	(0.91, 5.64)	2.40	(0.93, 6.22)
Hospitalization of acute myocardial infarction														
CKD stage 1	27	23600	1.14	1.00	(reference)	1.00	(reference)	30	23432	1.28	1.00	(reference)	1.00	(reference)
CKD stage 2	6	4828	1.24	0.94	(0.39, 2.27)	0.84	(0.35, 2.05)	3	4012	0.75	0.47	(0.14, 1.55)	0.45	(0.14, 1.46)
CKD stage 3	5	4372	1.14	0.90	(0.35, 2.34)	0.83	(0.31, 2.21)	2	4492	0.45	0.30	(0.07, 1.24)	0.28	(0.07, 1.18)
CKD stage 4	0	865	0.00	NA	-	NA	-	4	1027	3.89	2.51	(0.88, 7.13)	2.63	(0.90, 7.67)
CKD stage 5	3	624	4.81	3.60	(1.09, 11.88)*	3.24	(0.96, 10.93)	2	756	2.64	1.73	(0.41, 7.23)	1.61	(0.38, 6.86)

N: number of event; PY: person-year; IR: incidence rate per 1000 person-years; cHR: crude hazard ratio; aHR: adjusted hazard ratio; ^ꝉ^: adjusted for sex, age, comorbidites and medication, *: p-value<0.05; **: p-value<0.01; ***: p-value<0.001.

**Table 4b:** Association of different CKD stages with outcomes in the matched Cohort of MgO users.

	Without matching							1:1 PS matching						
Outcome	n	PY	IR	cHR	(95% CI)	aHR	(95% CI)	n	PY	IR	cHR	(95% CI)	aHR	(95% CI)
Acute kidney injury														
CKD stage 1	113	23066	4.90	1.00	(reference)	1.00	(reference)	118	22853	5.16	1.00	(reference)	1.00	(reference)
CKD stage 2	33	4695	7.03	1.16	(0.78, 1.70)	1.12	(0.76, 1.66)	24	3914	6.13	0.87	(0.56, 1.36)	0.90	(0.58, 1.40)
CKD stage 3	66	4048	16.3	2.79	(2.06, 3.78)***	2.16	(1.57, 2.98)***	54	4262	12.67	1.96	(1.42, 2.71)***	1.50	(1.07, 2.10)*
CKD stage 4	27	722	37.4	5.82	(3.82, 8.85)***	3.97	(2.55, 6.16)***	22	920	23.92	3.33	(2.11, 5.25)***	1.89	(1.17, 3.05)**
CKD stage 5	11	577	19.1	2.99	(1.61, 5.55)***	1.60	(0.84, 3.06)	21	651	32.27	4.41	(2.77, 7.01)***	2.38	(1.44, 3.93)***
Acute kidney disease														
CKD stage 1	449	21118	21.3	1.00	(reference)	1.00	(reference)	449	20862	21.52	1.00	(reference)	1.00	(reference)
CKD stage 2	138	4179	33.0	1.23	(1.01, 1.48)*	1.21	(1.00, 1.47)*	111	3548	31.28	1.06	(0.86, 1.31)	1.08	(0.87, 1.33)
CKD stage 3	178	3505	50.8	1.95	(1.64, 2.33)***	1.70	(1.41, 2.04)***	150	3770	39.79	1.45	(1.21, 1.75)***	1.20	(0.99, 1.46)
CKD stage 4	57	585	97.4	3.27	(2.48, 4.31)***	2.60	(1.96, 3.46)***	55	785	70.04	2.27	(1.72, 3.00)***	1.60	(1.19, 2.14)**
CKD stage 5	70	306	228.5	5.71	(4.44, 7.34)***	3.70	(2.82, 4.87)***	79	401	197.18	5.10	(4.02, 6.48)***	3.11	(2.39, 4.05)***
Hospitalization of acute kidney injury														
CKD stage 1	16	23682	0.68	1.00	(reference)	1.00	(reference)	23	23523	0.98	1.00	(reference)	1.00	(reference)
CKD stage 2	5	4846	1.03	1.55	(0.56, 4.28)	1.46	(0.52, 4.07)	3	4021	0.75	0.75	(0.22, 2.59)	0.79	(0.23, 2.72)
CKD stage 3	18	4349	4.14	5.62	(2.82, 11.23)***	4.23	(2.00, 8.93)***	8	4471	1.79	2.05	(0.90, 4.64)	1.51	(0.64, 3.54)
CKD stage 4	5	846	5.91	9.14	(3.33, 25.09)***	6.06	(2.06, 17.84)**	6	1027	5.84	6.68	(2.68, 16.62)***	4.41	(1.63, 11.93)**
CKD stage 5	1	632	1.58	2.45	(0.32, 18.54)	1.47	(0.18, 11.73)	4	745	5.37	6.20	(2.12, 18.13)***	3.73	(1.16, 12.04)*
Hospitalization of end-stage renal disease on dialysis														
CKD stage 1	196	23012	8.52	1.00	(reference)	1.00	(reference)	211	22801	9.25	1.00	(reference)	1.00	(reference)
CKD stage 2	24	4796	5.00	0.57	(0.37, 0.87)**	0.58	(0.38, 0.88)*	21	3986	5.27	0.53	(0.34, 0.84)**	0.56	(0.36, 0.88)*
CKD stage 3	61	4256	14.33	1.64	(1.23, 2.19)***	1.40	(1.03, 1.89)*	43	4414	9.74	1.01	(0.73, 1.40)	0.84	(0.59, 1.17)
CKD stage 4	62	729	85.06	9.40	(7.05, 12.53)***	6.72	(4.94, 9.13)***	56	913	61.34	6.07	(4.51, 8.17)***	3.88	(2.81, 5.34)***
CKD stage 5	106	207	510.97	44.50	(34.84, 56.86)***	27.90	(21.09, 37.12)***	100	356	281.04	24.10	(18.90, 30.74)***	13.50	(10.17, 17.92)***
Hospitalization of congestive heart failure with acute pulmonary edema														
CKD stage 1	9	23695	0.38	1.00	(reference)	1.00	(reference)	8	23538	0.34	1.00	(reference)	1.00	(reference)
CKD stage 2	1	4851	0.21	0.72	(0.09, 5.72)	0.59	(0.07, 4.82)	1	4023	0.25	1.02	(0.13, 8.30)	0.91	(0.11, 7.79)
CKD stage 3	3	4389	0.68	2.06	(0.56, 7.63)	1.72	(0.42, 7.03)	1	4497	0.22	0.81	(0.10, 6.50)	0.51	(0.06, 4.76)
CKD stage 4	0	865	0.00					2	1043	1.92	7.42	(1.56, 35.41)*	6.48	(1.12, 37.53)*
CKD stage 5	0	637	0.00					0	763	0.00				
Hospitalization of cardiac arrhythmia														
CKD stage 1	105	23302	4.51	1.00	(reference)	1.00	(reference)	94	23139	4.06	1.00	(reference)	1.00	(reference)
CKD stage 2	27	4759	5.67	1.42	(0.87, 2.31)	1.42	(0.87, 2.33)	23	3955	5.82	1.44	(0.84, 2.46)	1.50	(0.87, 2.57)
CKD stage 3	45	4168	10.80	2.23	(1.44, 3.44)***	2.01	(1.26, 3.20)**	41	4324	9.48	1.79	(1.11, 2.88)*	1.71	(1.03, 2.83)*
CKD stage 4	13	796	16.34	3.99	(2.05, 7.75)***	4.16	(2.08, 8.29)***	15	968	15.49	3.40	(1.74, 6.62)***	3.23	(1.59, 6.53)**
CKD stage 5	7	614	11.40	2.61	(1.05, 6.46)*	2.39	(0.93, 6.14)	7	735	9.53	2.27	(0.91, 5.64)	2.40	(0.93, 6.22)
Hospitalization of acute myocardial infarction														
CKD stage 1	27	23600	1.14	1.00	(reference)	1.00	(reference)	30	23432	1.28	1.00	(reference)	1.00	(reference)
CKD stage 2	6	4828	1.24	0.94	(0.39, 2.27)	0.84	(0.35, 2.05)	3	4012	0.75	0.47	(0.14, 1.55)	0.45	(0.14, 1.46)
CKD stage 3	5	4372	1.14	0.90	(0.35, 2.34)	0.83	(0.31, 2.21)	2	4492	0.45	0.30	(0.07, 1.24)	0.28	(0.07, 1.18)
CKD stage 4	0	865	0.00					4	1027	3.89	2.51	(0.88, 7.13)	2.63	(0.90, 7.67)
CKD stage 5	3	624	4.81	3.60	(1.09, 11.88)*	3.24	(0.96, 10.93)	2	756	2.64	1.73	(0.41, 7.23)	1.61	(0.38, 6.86)

N: number of event; PY: person-year; IR: incidence rate per 1000 person-years; cHR: crude hazard ratio; aHR: adjusted hazard ratio; ^ꝉ^: adjusted for sex, age, comorbidites and medication, *: p-value<0.05; **: p-value<0.01; ***: p-value<0.001.

## Data Availability

The data that support the findings of this study are available from the corresponding author upon reasonable request.
